# Unifying the analysis of continuous and categorical measures of weight loss and incorporating group effect: a secondary re-analysis of a large cluster randomized clinical trial using Bayesian approach

**DOI:** 10.1186/s12874-021-01499-0

**Published:** 2022-01-26

**Authors:** Fengming Tang, Christie A. Befort, Jo Wick, Byron J. Gajewski

**Affiliations:** 1 Department of Biostatistics & Data Science, University of Kansas Medical Center, Mail Stop 1026, 3901 Rainbow Blvd, Kansas City, KS 66160 USA; 2 Saint Luke’s Health System, Kansas City, MO 64111 USA; 3 Department of Population Health, University of Kansas Medical Center, Kansas City, KS 66160 USA

**Keywords:** Bayesian paradigm, Hierarchical model, Randomized clinical trial

## Abstract

**Background:**

Although frequentist paradigm has been the predominant approach to clinical studies for decades, some limitations associated with the frequentist null hypothesis significance testing have been recognized. Bayesian approaches can provide additional insights into data interpretation and inference by deriving posterior distributions of model parameters reflecting the clinical interest. In this article, we sought to demonstrate how Bayesian approaches can improve the data interpretation by reanalyzing the Rural Engagement in Primary Care for Optimizing Weight Reduction (REPOWER).

**Methods:**

REPOWER is a cluster randomized clinical trial comparing three care delivery models: in-clinic individual visits, in-clinic group visits, and phone-based group visits. The primary endpoint was weight loss at 24 months and the secondary endpoints included the proportions of achieving 5 and 10% weight loss at 24 months. We reanalyzed the data using a three-level Bayesian hierarchical model. The posterior distributions of weight loss at 24 months for each arm were obtained using Hamiltonian Monte Carlo. We then estimated the probability of having a higher weight loss and the probability of having greater proportion achieving 5 and 10% weight loss between groups. Additionally, a four-level hierarchical model was used to assess the partially nested intervention group effect which was not investigated in the original REPOWER analyses.

**Results:**

The Bayesian analyses estimated 99.5% probability that in-clinic group visits, compared with in-clinic individual visits, resulted in a higher percent weight loss (posterior mean difference: 1.8%[95% CrI: 0.5,3.2%]), a greater probability of achieving 5% threshold (posterior mean difference: 9.2% [95% CrI: 2.4, 16.0%]) and 10% threshold (posterior mean difference: 6.6% [95% CrI: 1.7, 11.5%]). The phone-based group visits had similar result. We also concluded that including intervention group did not impact model fit significantly.

**Conclusions:**

We unified the analyses of continuous (the primary endpoint) and categorical measures (the secondary endpoints) of weight loss with one single Bayesian hierarchical model. This approach gained statistical power for the dichotomized endpoints by leveraging the information in the continuous data. Furthermore, the Bayesian analysis enabled additional insights into data interpretation and inference by providing posterior distributions for parameters of interest and posterior probabilities of different hypotheses that were not available with the frequentist approach.

**Trial registration:**

ClinicalTrials.gov Identifier NCT02456636; date of registry: May 28, 2015.

**Supplementary Information:**

The online version contains supplementary material available at 10.1186/s12874-021-01499-0.

## Introduction

Although frequentist paradigm has been the predominant approach to clinical studies in the past several decades and we have seen tremendous progress in medicine, some limitations associated with the frequentist null hypothesis significance testing (NHST) that reports dichotomized *p* values have been recognized in statistic society [1, 2]. One of the important problems with NHST is that *p* values are very prone to misinterpretation and are often misused in medical studies [3]. The most common misinterpretation of *p* values is the probability of the null hypothesis. Frequentist methods do not estimate the probability of hypotheses and a *p* value is the probability of observing data as extreme or more extreme if the null hypothesis is true (no treatment effect), which may not be of the researcher’s interest. Additionally, *p* values are routinely dichotomized using a predefined *α* level (usually 0.05) to facilitate medical decision-making. A nonsignificant *p* value (> 0.05) is sometimes misinterpreted as ‘no effect’ while a nonsignificant result does not distinguish between a true null effect and a lack of statistic power [4]. When the sample size is small or when the variation is big, *p* values can be big even when there is a true effect. Bayesian approaches, on the other hand, can provide additional in-depth insights into data interpretation by deriving posterior distributions of model parameters reflecting clinical interests. The probabilities of different hypotheses can be estimated from the posterior distributions of model parameters, e.g., the probability of treatment A better than treatment B, or the probability of treatment A equivalent to treatment B, etc. This allows one to make probabilistic interpretations according to the entire posterior distributions. Furthermore, Bayesian approaches are also extremely flexible in that the posterior distributions can be converted to metrics of clinical interests without having to use extra modeling. In this article, we focused on demonstrating how Bayesian approaches can improve interpretation by reanalyzing the REPOWER [5] data using Bayesian models. We aim to accomplish three goals for weight loss clinical trials: (1) encourage posterior probabilities for interpretation; (2) harmonize clinical weight loss metrics for percent weight loss (continuous) and achievement of weight loss clinical thresholds (binary); and (3) model the clustering of the partially nested intervention group effect common in weight loss studies but ignored in the original REPOWER paper.

Obesity is a chronic condition affecting an increasing number of Americans with the prevalence reaching 42% in 2017–2018 [6]. It is a serious health risk and is associated with a wide range of morbidities [7]. The Centers for Medicare and Medicaid Services (CMS) approved to cover Intensive Behavioral Therapy for Obesity (IBT) with up to 22 individual 15-min face-to-face visits over a 12-month period in 2011 [8]. The CMS employs a fee-for-service delivery model which has been challenged and questioned. A variety of care delivery models have arisen in addition to the traditional face-to-face office visit. REPOWER [5] is a cluster randomized clinical trial comparing the fee-for-service individual delivery model to two alternatives: in-clinic group visits and phone-based group visits. Participant weight was measured at baseline, 6, 18, and 24 months by trained staff. The primary endpoint was weight loss at 24 months. The secondary endpoints included the proportions of participants achieved 5 and 10% weight loss at 24 months.

In the original analyses [5], frequentist methods were used and inferences were drawn based on *p* values and confidence intervals. For the primary endpoint, a linear mixed model was used. The in-clinic group visits, but not the phone-based group, resulted in a statistically significantly higher weight loss at 24 months when compared with the in-clinic individual visits. For the secondary endpoints, two separate mixed effect logistic models were used to compare the proportions of participants of achieving 5 and 10% weight loss at 24 months. None of the comparisons resulted in a significant *p* value. In this article, we reanalyzed the percent weight loss over time using a Bayesian hierarchical model with noninformative priors. We first obtained the posterior distributions of weight loss at 24 months for each arm using Hamiltonian Monte Carlo. We then estimated the probabilities of having a greater weight loss in the in-clinic group visits and the phone-based group visits vs. the in-clinic individual visits. With the same model, we also obtained the posterior distributions for the probabilities of achieving 5% (or 10%) weight loss in each arm and the probabilities of having greater probabilities of achieving the weight loss thresholds in the two group-based arms vs. the in-clinic individual visits. The Bayesian approach not only provided a better interpretation by reporting probabilities of different hypotheses, but also unified the analyses of the continuous (the primary endpoint) and categorical measures of weight loss (the secondary endpoints) using a single model. This approach resulted in consistent inferences for different endpoints and achieved higher power for the secondary endpoints in comparison with the original analyses.

Moreover, the original analyses took into consideration the clustering of sites but ignored the clustering of intervention group in the two group-based arms. Intervention group was partially nested because it was relevant to the two group-based arms only. The Bayesian approach can easily handle complex problems using the same statistical framework. We used a four-level hierarchical model with an additional level to assess the partially nested group assignment on the effect of delivery models.

## Methods

### Study design and data structure

REPOWER is a cluster randomized clinical trial with thirty six primary practices from three affiliations (academic medical centers that recruited participants for the study: the University of Kansas Medical Center (KUMC), the University of Nebraska Medical Center (UNMC), and the Marshfield Clinic in Wisconsin (Marshfield clinic)) randomly assigned to one of the three study arms in equal numbers: 1) in-clinic individual visits in which the participants received 15-min face-to-face individual counseling sections; 2) in-clinic group visits in which the participants received group visits held at practices with a median of 14 participants per group; 3) Phone-based group visits in which participants received lifestyle intervention delivered remotely via audio-only conference calls with a median of 14 participants per group. The trial was approved by institutional review boards at the University of Kansa Medical Center and the VA Nebraska-Western Iowa Health Care System. All participants provided written informed consent. The re-analysis was done on deidentified data. 1407 participants were included in the final analysis. Weight was measure at baseline, 6, 18, and 24 months by trained staff. The primary outcome was weight loss at 24 months. The secondary outcomes included the proportions of achieving 5 and 10% weight loss at 24 months. The detailed information about the trial conduction has been published by Befort et al. [5]. In this article, we first analyzed the percent weight loss using a three-level Bayesian hierarchical model to compare the effect of different intervention delivery models on percent weight loss. A second Bayesian hierarchical model additionally included intervention group as a partially nested effect to assess its effect on weight loss.

### Model 1: three level Bayesian hierarchical model for percent weight loss

Let *y*_*ijt*_ be the percent weight loss for participant *j* from site *i* at time *t*. *x*_1_ and *x*_2_ are the arm indicators: (0,0) for in-clinic individual visits, (1,0) for in-clinic group visits, and (0,1) for phone-based group visits. *t*_18_ and *t*_24_ are the time indicators: (0,0) for month 6, (1,0) for month 18, and (0,1) for month24. We also include arm and time interactions so that delivery model effect can be evaluated at each time point. To be consistent with the original analyses, we included affiliation indicators as covariates (denoted by *x*_3_ *and x*_4_). The three-level Bayesian hierarchical model can be represented as follows.

$${y}_{ijt}={\alpha}_{0 ij}+{\beta}_1{x}_1+{\beta}_2{x}_2+{\beta}_3{t}_{18}+{\beta}_4{t}_{24}+{\beta}_5{x}_1\ast {t}_{18}+{\beta}_6{x}_1\ast {t}_{24}+{\beta}_7{x}_2\ast {t}_{18}+{\beta}_8{x}_2\ast {t}_{24}+{\upbeta}_9{x}_3+{\beta}_{10}{x}_4+{\epsilon}_{ijt}$$

- *α*_0*ij*_ = *α*_0*i*0_ + *γ*_*j*_, where ${\gamma}_j\sim N\left(0,{\sigma}_{\gamma}^2\right)$ is patient level variation.
- *α*_0*i*0_ = *α*_000_ + *η*_*i*_, where *η*_*i*_ ~ $N\left(0,{\sigma}_{\eta}^2\right)$ is site level variation and *a*_000_ is the model intercept.
- *ϵ*_*ijt*_~N(0, *σ*^2^) is within patient residual error.

Noninformative priors were used to make like to like comparison with the frequentist analyses: Stan default flat prior, uniform distribution on the real line, was used for *a*_000_ and *βs*; truncated normal distribution *N*^+^(0, 10) was used for the standard deviations (*σ*, *σ*_*γ*_, and *σ*_*η*_ ) to ensure only positive values were allowed.

### Model 2: Bayesian hierarchical model for percent weight loss with group assignment as a partially nested effect

Participants in the in-clinic group visits arm and the phone-based group visits arm received the interventions in groups. We wanted to examine the impact of group assignment on the effect of intervention delivery methods for the two group-based arms, which was not tackled in the original analyses. In model 2, we utilized a four-level hierarchical Bayesian model with the group assignment as a partially nested effect to assess the effect of intervention group.

Let *k* > 0 index the intervention group for participants in the two group-based arms. For participants in the in-clinic individual visits arm, *k* = 0. The four-level Bayesian hierarchical model can be represented as follows.

$${y}_{ikjt}={\alpha}_{0 ikj}+{\beta}_1{x}_1+{\beta}_2{x}_2+{\beta}_3{t}_{18}+{\beta}_4{t}_{24}+{\beta}_5{x}_1\ast {t}_{18}+{\beta}_6{x}_2\ast {t}_{18}+{\beta}_7\ast {t}_{24}+{\beta}_8{x}_2\ast {t}_{24}+{\upbeta}_9{x}_3+{\beta}_{10}{x}_4+{\epsilon}_{ikjt}$$

- *α*_0*ikj*_ = *α*_0*ik*0_ + *γ*_*j*_, where ${\gamma}_j\sim N\left(0,{\sigma}_{\gamma}^2\right)$ represents the patient level variation.
- *α*_0*ik*0_ = *α*_0*i*00_ + *ϑ*_*k*_, where ${\vartheta}_k\sim N\left(0,{\sigma}_{\vartheta}^2\right)$ represents the intervention group level variation for participants in the two group-based arms and for participants in the in-clinic individual arm *ϑ*_0_ = 0.
- *α*_0*i*00_ = *α*_0000_ + *η*_*i*_, *η*_*i*_ ~ $N\left(0,{\sigma}_{\eta}^2\right)$ represents the site level variation and *a*_0000_ is the intercept.
- ϵ_ikjt_~N(0, σ^2^) is the within patient residual error

The same noninformative priors as in Model 1 were used. To assess whether including intervention group as an additional hierarchical level improved model fit, we used two model selection methods to compare Model 1 and Model 2: leave-one-out cross-validation (Loo-CV) and widely available information criterion (WAIC) [9]. Both methods are implemented in the *loo* R package [10].

### Quantities of interest

The quantities representing the expected 24 months percent weight loss for participants from the three affiliations in the in-clinic individual arm are Δ_1 _ 1_ = *a*_000_ + *β*_4_, Δ_1 _ 2_ = *a*_000_ + *β*_4_ + *β*_9_, and Δ_1 _ 3_ = *a*_000_ + *β*_4_ + *β*_10_, respectively. We use the arithmetic average ${\Delta}_1=\frac{\Delta_{1\_1}+{\Delta}_{1\_2}+{\Delta}_{1\_3}}{3}={a}_{000}+{\beta}_4+\frac{1}{3}{\beta}_9+\frac{1}{3}{\beta}_{10}$ to represent the average expected percent weight loss for the in-clinic individual arm. Similarly, for in-clinic group visits and phone-based group, the average expected 24 months percent weight loss are ${\Delta}_2={a}_{000}+{\beta}_1+{\beta}_4+{\beta}_6+\frac{1}{3}{\beta}_9+\frac{1}{3}{\beta}_{10}$ and ${\Delta}_3={a}_{000}+{\beta}_2+{\beta}_4+{\beta}_8+\frac{1}{3}{\beta}_9+\frac{1}{3}{\beta}_{10}$ respectively. Their posterior distributions can be obtained from the MCMC samples of *a*_000_ and *β* ′ *s*. The absolute differences in 24 months percentage weight loss in comparison to the in-clinic individual visits can be assessed using *δ*_2_ = Δ_2_ − Δ_1_ = *β*_1_ + *β*_6_ for the in-clinic group arm and *δ*_3_ = Δ_3_ − Δ_1_ = *β*_2_ + *β*_8_ for the phone-based group arm. The probabilities of having a higher weight loss can be evaluated using the proportions of the corresponding MCMC samples greater than 0.

Additionally, the posterior predictive distribution for the probability of achieving 5% or 10% threshold can be obtained using MCMC samples of model parameters. Let *z*_1_ be the 24 months percent weight loss for a new participant in the in-clinic individual arm. It follows a $N\left({\Delta}_1,{\sigma}^2+{\sigma}_r^2+{\sigma}_{\eta}^2\right)$ conditional on model parameters ${\boldsymbol{\theta}}_1=\left\{{\Delta}_1,{\sigma}^2,{\sigma}_r^2,{\sigma}_{\eta}^2\right\}$. The posterior predictive distribution of *z*_1_ is therefore ∫ϕ(*z*_1_| ***θ***_**1**_)*p*(***θ***_1_| ***y***)*d****θ***_**1**_, where ϕ(*z*_1_| ***θ***_**1**_) is the normal probability density function and *p*(***θ***_1_| ***y***) is the posterior distribution of ***θ***_**1**_. The posterior predictive distribution for the probability of achieving 5% threshold is ${\int}_5^{\infty}\int \upphi \left({z}_1|{\boldsymbol{\theta}}_{\mathbf{1}}\right)p\left({\boldsymbol{\theta}}_1|\boldsymbol{y}\right)d{\boldsymbol{\theta}}_{\mathbf{1}}d{z}_1,$ which is equivalent to $\int {\int}_5^{\infty}\upphi \left({z}_1|{\boldsymbol{\theta}}_{\mathbf{1}}\right)d{z}_1p\left({\boldsymbol{\theta}}_1|\boldsymbol{y}\right)d{\boldsymbol{\theta}}_{\mathbf{1}}$ and its posterior MCMC samples can be obtained by evaluating ${\int}_5^{\infty}\upphi \left({z}_1|{\boldsymbol{\theta}}_{\mathbf{1}}\right)d{z}_1$ at each MCMC samples of the model parameters *α*_000_, *βs*, and *σs*. Similarly, the posterior predictive distribution of probability of achieving 5% threshold for the in-clinic group arm and phone-based group arm can be obtained by MCMC samples of ${\int}_5^{\infty}\upphi \left({z}_2|{\boldsymbol{\theta}}_2\right)d{z}_2$ and ${\int}_5^{\infty}\upphi \left({z}_3|{\boldsymbol{\theta}}_2\right)d{z}_3$ respectively, where ${\boldsymbol{\theta}}_2=\left\{{\Delta}_2,{\sigma}^2,{\sigma}_r^2,{\sigma}_{\eta}^2\right\}$ and ${\boldsymbol{\theta}}_3=\left\{{\Delta}_3,{\sigma}^2,{\sigma}_r^2,{\sigma}_{\eta}^2\right\}$. The posterior predictive distributions of the probabilities of achieving 10% weight loss at 24 months can be obtained by simply changing the lower integration bound to 10.

### Computation and software

Hamiltonian Monte Carlo [11] was performed in Stan [12] to obtain the posterior distributions for parameters of interest. Figure representations of posterior distributions were computed from gaussian kernel density estimates, which provided a smoothed version of the sampled histograms. R package *Rstan* was used as the interface to call Stan code [13]. All the other analyses and plots were conducted in R. The Stan code for the two models can be found in the Additional file 1.

## Results

### Model convergence assessment and predictive checking

For both models we ran four parallel MCMC chains with starting points randomly generated from the prior distributions. For each chain, we allowed 3000 iterations for the sampler to converge and another 3000 for sampling the posterior distributions. Convergence was checked visually utilizing trace plots. We also checked the potential scale reduction factor [14] and the effective sample size. For all model parameters, $\hat{R}$ was less than 1.01 and effective sample size was > 400.

### Model result

#### Model 1

Table 1 summarizes the model parameters using posterior means and 95% credible intervals (CrI, calculated by taking the 2.5 and 97.5 percentiles of the posterior distributions) based on their MCMC samples of the posterior distributions. Because non-informative priors were used, the means and 95% CrIs were very close to the result from the original linear mixed-effect multilevel model.

**Table 1:** Posterior means and 95% credible intervals for model parameters in Model 1

		Mean	Standard Deviation	2.50%	97.50%
Intercept	*a*_000_	6.24	0.54	5.18	7.29
In-clinic group effect (ref: in-clinic indiv)	*β*_1_	2.67	0.69	1.36	4.04
Phone-based group effect (ref: in-clinic indiv)	*β*_2_	1.98	0.69	0.64	3.34
18 months effect (ref: 6 months)	*β*_3_	−2.26	0.28	−2.8	−1.72
24 months effect (ref: 6 months)	*β*_4_	−3.05	0.28	−3.6	− 2.52
In-clinic group effect * 18 months effect	*β*_5_	0.06	0.61	−1.15	1.26
Phone-based group effect *18 months effect	*β*_6_	−2.16	0.79	−3.7	− 0.62
In-clinic group effect * 24 months effect	*β*_7_	−0.41	0.38	−1.16	0.34
Phone-based group effect * 24 months effect	*β*_8_	−0.1	0.4	−0.87	0.68
Affiliation: Marshfield Clinic (ref: KUMC)	*β*_9_	−0.83	0.39	−1.58	−0.07
Affiliation: UNMC (ref: KUMC)	*β*_10_	−0.51	0.39	−1.28	0.28
Observation level variation	*σ*	3.92	0.06	3.8	4.03
Site level variation	*σ*_*η*_	0.98	0.36	0.22	1.67
Patient level variation	*σ*_*r*_	6.66	0.15	6.38	6.95

**Fig. 1:**
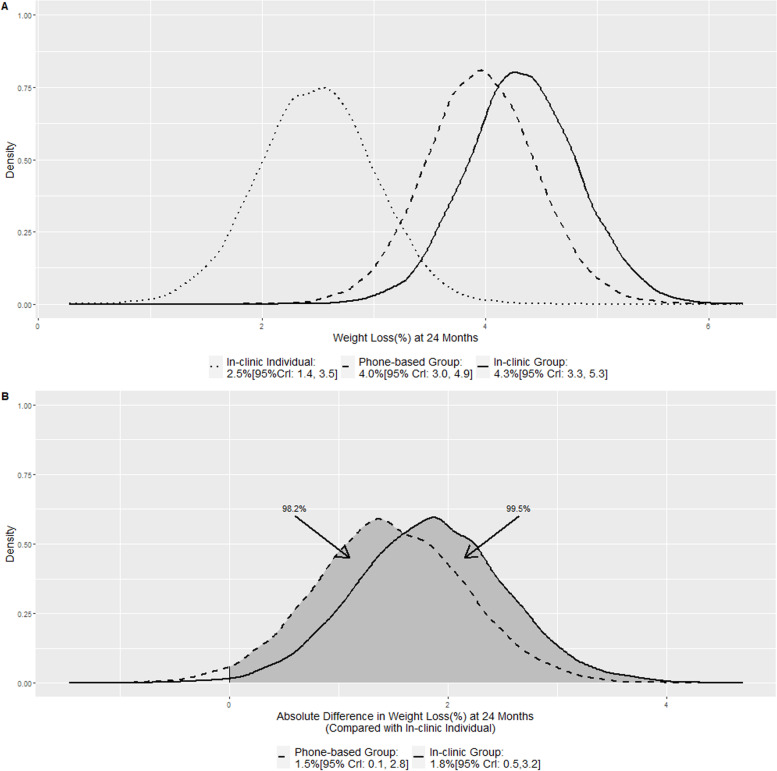
Posterior distributions of the expected weight loss(%) (**A**) and posterior distributions of the absolute difference in weight loss(%) when compared with in-clinic individual visits (**B**) at 24 months

Figure 1A displays the posterior distribution of the expected 24 months weight loss for the three arms: in-clinic individual visits (Δ_1_), in-clinic group visits (Δ_2_), and phone-based group visits (Δ_3_). The corresponding posterior means and credible intervals were 2.5%[95% CrI: 1.4, 3.5], 4.3[95% CrI: 3.3, 5.3], and 4.0%[95% CrI: 3.0, 4.9], respectively. They were almost identical to the estimated means and confidence intervals reported in the original analysis: 2.5%[95%CI: 1.4, 3.5], 4.3[95% CI: 3.3, 5.3], and 3.8[95% CI: 2.8,4.9], respectively.

Figure 1B displays the posterior distributions of the absolute difference in the expected 24 months percent weight loss for the in-clinic group visits (*δ*_2_) and the phone-based group visits (*δ*_3_) when compared with the in-clinic individual visits. The corresponding posterior means and 95% credible intervals were 1.8% [95% CrI: 0.5,3.2] and 1.5% [95% CrI: 0.1, 2.8] respectively. The shaded areas to the right of zero represent the probabilities of having a greater weight loss: 99.5 and 98.2% respectively. The original analyses reported there was a significant difference between the in-clinic group visits (1.8% [95% CI: 0.4, 3.2; *p* value: 0.01]), but not in the phone-based visits (1.3[95% CI: − 0.1, 2.8; *p* value: 0.06]) because the *p* value was slightly bigger than 0.05.

**Fig. 2:**
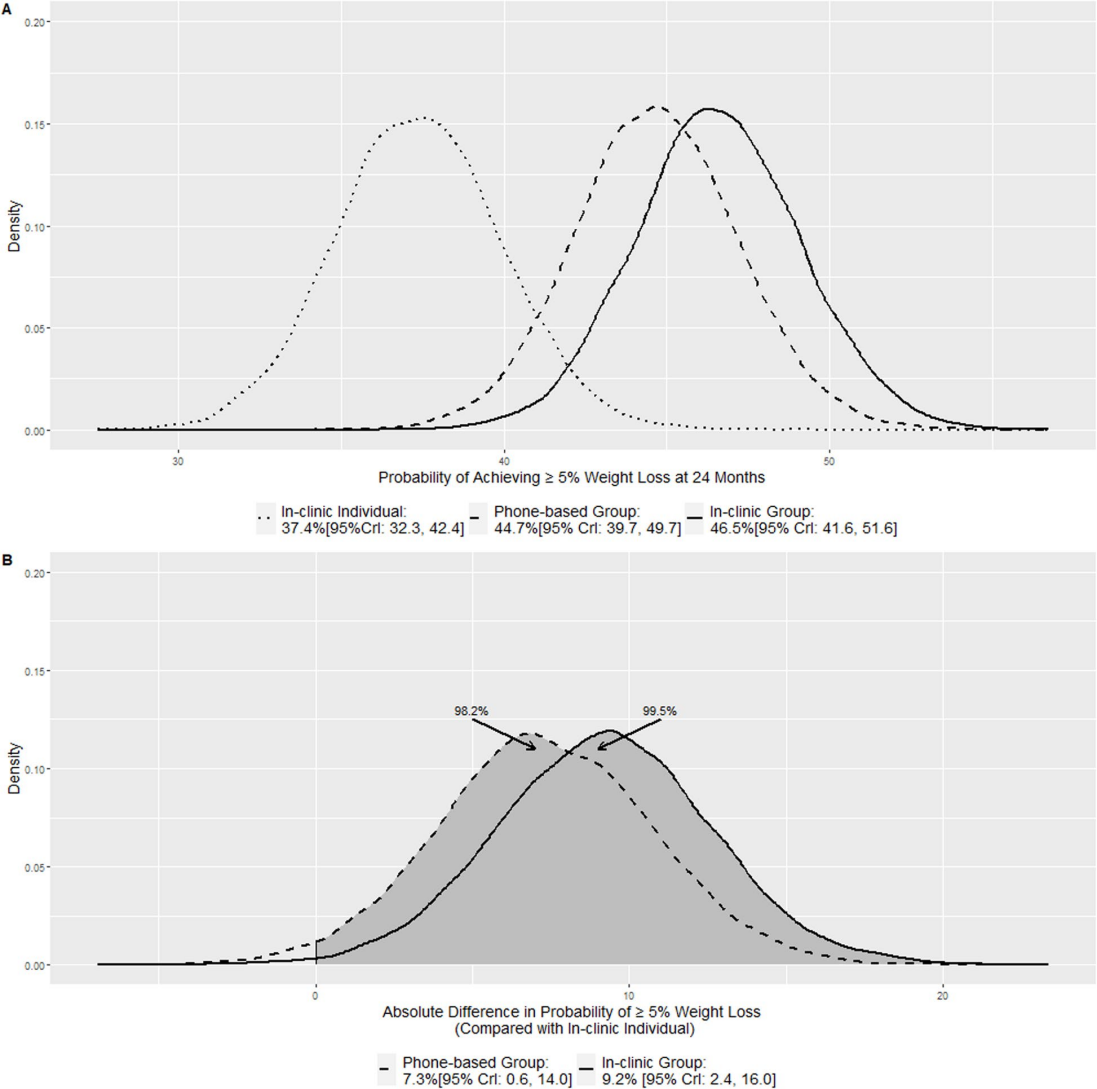
Posterior distributions of the probability of achieving 5% weight loss (**A**) and Posterior distributions of the absolute difference in the probability of achieving 5% weight loss when compared with in-clinic individual visits (**B**)

**Fig. 3:**
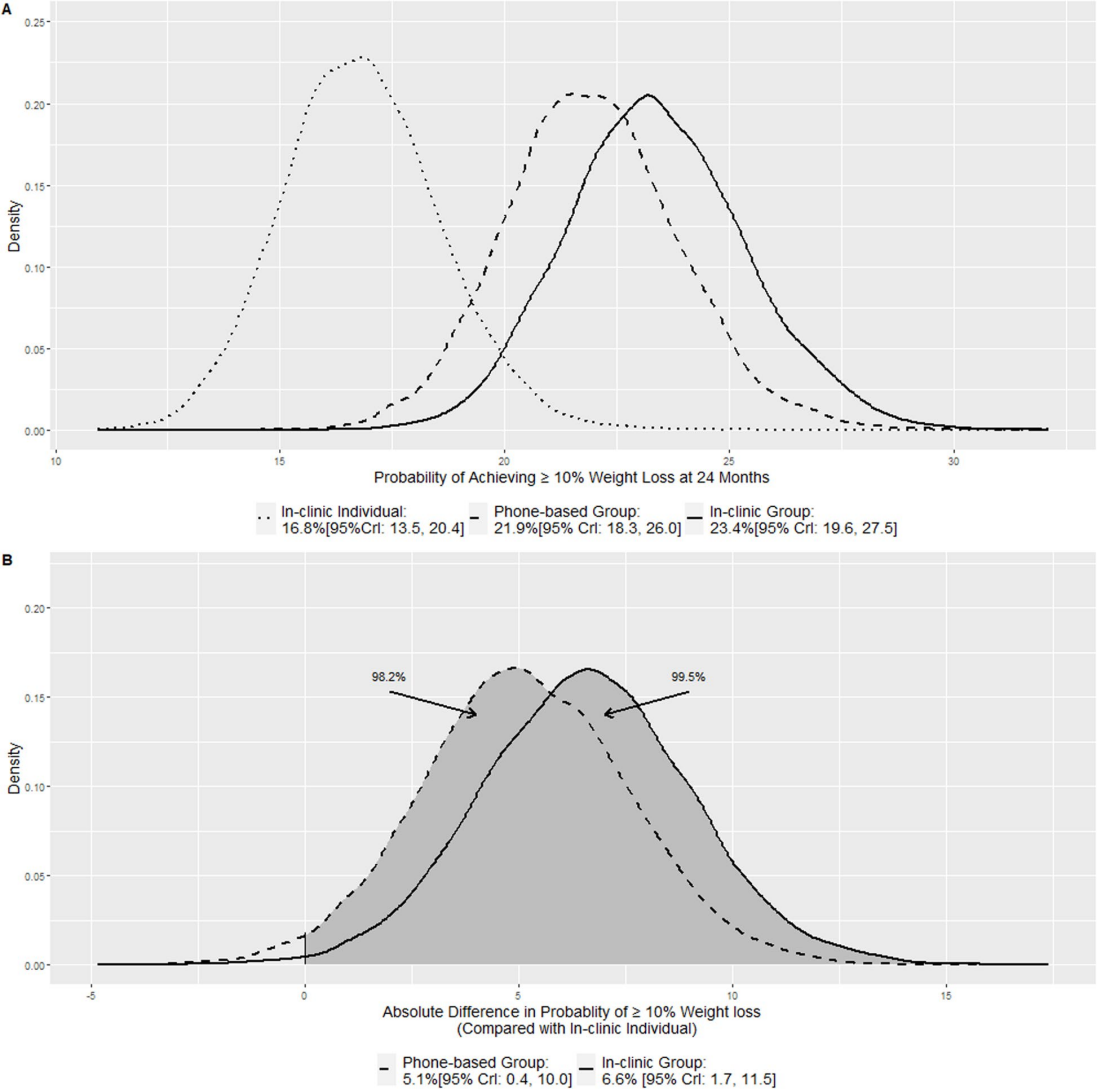
Posterior distributions of the probability of achieving 10% weight loss (**A**) and Posterior distributions of the absolute difference in the probability of achieving 10% weight loss when compared with in-clinic individual visits (**B**)

Figures 2A and 3A display the posterior distributions for the probabilities of achieving 5 and 10% 24 months weight loss respectively. The shapes of the three density plots were very similar to those in Fig. 1A due to the relationship between the probabilities of achieving weight loss threshold and Δ_1_, Δ_2_, and Δ_3_ illustrated in the section Quantities of interest. In the order of in-clinic individual visits, in-clinic group visits, and phone-based group visits, the posterior mean and the 95% credible interval were 37.4%[95% CrI: 32.3, 42.4], 46.5%[95% CrI: 41.6, 51.6], and 44.7%[95% CrI: 39.7, 49.7] for achieving 5% threshold; 16.8%[95% CrI: 13.5, 20.4], 23.4%[95% CrI: 19.6, 27.5], and 21.9%[95% CrI: 18.1, 26.0] for achieving 10% threshold. In the original analyses, two separate mixed effect logistic models were used to estimate proportions of 5 and 10% weight loss: 36.0% [95% CI:30.2, 42.3], 44.1% [95% CI: 35.2, 47.8], and 41.4% [95% CI: 37.9, 50.6] for 5% threshold, and 17.1% [95% CI: 13.3, 21.8], 22.6% [95% CI: 18.1, 27.9], and 22.3% [95% CI: 17.9, 27.6] for 10% threshold. While the Bayesian point estimates for proportions of achieving 10 and 5% weight loss were close to the original result, the interval widths were narrower in the Bayesian model because it leveraged the continuous model.

Figures 2B and 3B display the absolute differences in the probabilities of achieving 24 months weight loss thresholds for the in-clinic group visits and the phone-based group visits when compared with the in-clinic individual visits: 9.2% [95% CrI: 2.4, 16.0] and 7.3%[95% CrI: 0.6, 14.0] respectively for achieving 5% threshold, and 6.6% [95% CrI: 1.7, 11.5] and 5.1%[95% CrI: 0.4, 10.0] respectively for achieving 10% threshold. The shaded areas (to the right of zero) represent the probabilities of having a higher probability of achieving the thresholds. For both 5 and 10% weight loss, the probabilities were 99.5% for in-clinic group arm and 98.2% for the phone-based group arm and they were consistent with the probabilities of having a greater weight loss than the in-clinic individual visits arm as shown in Fig. 1B. In the original analyses, the odds ratios of achieving the thresholds were reported for the in-clinic group visits and the phone-based group visits: 1.4 [95% CI: 1.0, 2.0; *p* value: 0.07] and 1.3 [95% CI: 0.9, 1.8; *p* value: 0.22] respectively for 5% threshold, and 1.4 [95% CI: 0.9, 2.1; *p* value: 0.09] and 1.4 [95% CI: 0.9, 2.1; *p* value: 0.11] respectively for 10% threshold. The authors concluded there was no significant difference for both threshold and for both in-clinic group vs. in-clinic individual and phone-based group vs. in-clinic individual comparisons.

#### Model 2

Table 2 shows the posterior means and 95% credible intervals for model parameters in Model 2 based on their MCMC samples of the posterior distributions. The values were very close to Model 1 for the parameters in common. The mean and 95% CrIs for *σ*_*ϑ*_ were 1.28 [95% CrI: 0.28, 2.08]. Both Looic and WAIC were slightly bigger in Model 1 (Table 3): 22025 vs. 22,016 and 21,816 vs. 21,814, respectively. The differences were small in comparison with their standard error: 8.4 (4.6) and 2.4 (3.0). We concluded that Model 2 did not improve model fit significantly. All conclusions drawn in Model 1 held in Model 2.

**Table 2:** Posterior mean and 95% credible interval for model parameters in Model 2

		Mean	Standard deviation	2.50%	97.50%
Intercept	*a*_000_	6.25	0.53	5.2	7.28
In-clinic group effect (ref: in-clinic indiv)	*β*_1_	2.67	0.7	1.31	4.05
Phone-based group effect (ref: in-clinic indiv)	*β*_2_	1.94	0.7	0.53	3.34
18 months effect (ref: 15 months)	*β*_3_	−2.26	0.28	−2.8	− 1.71
24 months effect (ref: 15 months)	*β*_4_	−3.06	0.28	−3.61	− 2.51
In-clinic group effect * 18 months effect	*β*_5_	0.09	0.62	−1.12	1.32
Phone-based group effect * 18 months effect	*β*_6_	−2.13	0.81	−3.73	−0.55
In-clinic group effect * 24 months effect	*β*_7_	−0.41	0.39	−1.19	0.35
Phone-based group effect * 24 months effect	*β*_8_	−0.1	0.39	−0.88	0.66
Affiliation: Marshfield Clinic (ref: KUMC)	*β*_9_	−0.83	0.39	−1.59	−0.06
Affiliation: UNMC (ref: KUMC)	*β*_10_	−0.51	0.39	−1.27	0.26
Observation level variation	*σ*	3.92	0.06	3.81	4.03
Site level variation	*σ*_*η*_	0.94	0.37	0.15	1.66
Group level variation	*σ*_*ϑ*_	1.28	0.44	0.28	2.08
Patient level variation	*σ*_*r*_	6.59	0.15	6.29	6.89

**Table 3:** Leave-one-out cross validation (loo-cv) and widely available information criterion (WAIC) for Model 1 and Model 2

	Looic (se)	WAIC (se)
Model 1	22,025.1 (127.4)	21,816.1 (122.6)
Model 2	22,016.7 (126.2)	21,813.7 (122.2)
Model 1 – Model 2	8.4 (4.6)	2.4 (3.0)

## Conclusion and discussion

Frequentist analyses base inferences on *p* values and confidence intervals. *P* values are not the probability of null hypotheses and heavily depends on the sample size and the variation of the endpoints. The decision-making using dichotomized *p* values is not as objective as some researchers believe. A *p* value of 0.06 and 0.01 are not very different, yet when using the threshold *α* = 0.05, a *p* value of 0.06 indicates a nonsignificant result and a *p* value of 0.01 indicates a significant result. For example, the original analyses concluded, when compared to in-clinic individual visit, there was a significantly greater weight loss at 24 months for the in-clinic group visits (*p* value: 0.01), but not for phone-based group visits (*p* value: 0.06). Conversely, the current Bayesian analysis reported that the probability of with a greater weight loss in the in-clinic group visits and phone-based group visits were 99.5 and 98.2% respectively, from which we concluded that both group-based arms were superior than the in-clinic individual visits with high confidence.

For the secondary endpoints, the original analyses used two separate mixed effect logistic regressions to compare the odds of achieving 5 and 10% weight loss. Studies have shown that dichotomizing continuous endpoints results in a loss of information and reduced power [15–17]. The current Bayesian analysis assessed the probabilities achieving 5 and 10% weight loss by integrating the posterior predictive distributions of the weight loss and reported 99.5 and 98.2% respectively while the original analyses reported there were no significant differences across the board. Furthermore, the Bayesian analysis also provided the absolute differences in probabilities of achieving 5 and 10% weight loss in the in-clinic group visits and phone-based group visits vs. the in-clinic individual visits, which may be preferred by clinicians than odds ratios reported in the original analysis.

In the Quantities of interest section, we used arithmetic average across affiliations to obtain the average expected percent weight loss for each arm. This method gives each affiliation the same weight. There are other choices for the averaging weights, e.g., weights that are proportionate to the numbers of participants or the numbers of sites in each affiliation. The method to use should be determined by the inference one intends to make. For the current study, the primary goal was to compare the three treatment arms. When the proportions of patients in each affiliation are similar across the three arms, the method would not affect the conclusion because *β*_9_ and *β*_10_ will be cancelled out when we take the difference between arms. Therefore, we would reach the same conclusion if we use different weights that are proportionate to the numbers of participants in each affiliation. Besides the advantages we discussed in this study, Bayesian approaches have other strengths including the ability to incorporate previous evidence through prior distributions to inform the posterior distributions and the ability to update the posterior distributions when new evidences emerge. Bayesian approaches have gained popularity in recent years owing to the advancement in powerful computing capacity and the invention of efficient Bayesian statistical software. However, Bayesian approaches remain underused and are often used as secondary re-analyses. We hope to see Bayesian approaches being adopted more frequently as primary analysis in clinical studies.

## Supplementary Information

**Additional file 1.**

## Data Availability

Data will be made available upon approved requests sent to cbefort@kumc.edu.
