# Supplementary material for: Unifying the analysis of continuous and categorical measures of weight loss and incorporating group effect: a secondary re-analysis of a large cluster randomized clinical trial using Bayesian approach
Source: BMC Med Res Methodol. 2022 Jan 26;22:28. doi: 10.1186/s12874-021-01499-0 (PMC8790853; doi:10.1186/s12874-021-01499-0)
Supplement: Supplementary file 1 — Additional file 1. [file 12874_2021_1499_MOESM1_ESM.docx]

# Appendix

**Stan code for model 1**

data {

int<lower=1> N; // total number of observations

vector[N] Y; // response variable

int<lower=1> K; // number of fixed effects

matrix[N, K] X; // design matrix

int<lower=1> N_1; // number of sites

int<lower=1> M_1; // number of site level coefficients

int<lower=1> J_1[N]; // site indicators

vector[N] Z_1_1; //site level predictor values

int<lower=1> N_2; // number of patients

int<lower=1> M_2; // number of patient level coefficients

int<lower=1> J_2[N]; // patient indication

vector[N] Z_2_1; //patient level predictor values

//predictive data

int<lower=1> N_tilde;

matrix[N_tilde, (K-1)] X_tilde;

}

transformed data {

int Kc = K - 1;

matrix[N, Kc] Xc; // centered version of X without an intercept

vector[Kc] means_X; // column means of X before centering

for (i in 2:K) {

means_X[i - 1] = mean(X[, i]);

Xc[, i - 1] = X[, i] - means_X[i - 1];

}

}

parameters {

vector[Kc] b; // fixed effects

real Intercept; // temporary intercept for centered predictors

real<lower=0> sigma; // residual SD

vector<lower=0>[M_1] sd_1; //site level standard deviations

vector[N_1] z_1[M_1]; // standardized site level effects

vector<lower=0>[M_2] sd_2; // participant standard deviation

vector[N_2] z_2[M_2]; // standardized participant effects

}

transformed parameters {

vector[N_1] r_1_1; // actual site level effects

vector[N_2] r_2_1; // actual participant level effects

vector[N] mu = Intercept + rep_vector(0.0, N);

r_1_1 = (sd_1[1] * (z_1[1]));

r_2_1 = (sd_2[1] * (z_2[1]));

for (n in 1:N) mu[n] += r_1_1[J_1[n]] * Z_1_1[n] + r_2_1[J_2[n]] * Z_2_1[n];

}

model {

// likelihood including all constants

target += normal_id_glm_lpdf(Y | Xc, mu, b, sigma);

target += normal_lpdf(sigma | 0, 10);

target += normal_lpdf(sd_1 | 0,10);

target += std_normal_lpdf(z_1[1]);

target += normal_lpdf(sd_2 | 0,10);

target += std_normal_lpdf(z_2[1]);

}

generated quantities {

// actual population-level intercept

real b_Intercept = Intercept - dot_product(means_X, b);

vector[N_tilde] y_tilde ;

vector[N_tilde] y_pred ;

vector [N_tilde] prob_5;

vector [N_tilde] prob_10;

vector [N] log_lik;

for (i in 1:N) {

log_lik[i] =normal_lpdf(Y[i] |mu[i] + Xc[i]*b, sigma);

}

for (i in 1:N_tilde) {

y_tilde[i]=normal_rng(b_Intercept+X_tilde[i]*b, sqrt(sd_1[1]^2+sd_2[1]^2+sigma^2));

y_pred[i]=b_Intercept+X_tilde[i]*b;

prob_5[i] = 1-normal_cdf(5, b_Intercept+X_tilde[i]*b, sqrt(sd_1[1]^2+sd_2[1]^2+sigma^2));

prob_10[i] = 1-normal_cdf(10, b_Intercept+X_tilde[i]*b, sqrt(sd_1[1]^2+sd_2[1]^2+sigma^2));

}

}

**Stan code for model 2**

data {

int<lower=1> N; // total number of observations

vector[N] Y; // response variable

int<lower=1> K; // number of fixed effects

matrix[N, K] X; // design matrix

int<lower=1> N_1; // number of sites

int<lower=1> M_1; // number of site level coefficients

int<lower=1> J_1[N]; //site indicators

vector[N] Z_1_1; //site level predictor values

int<lower=1> N_2; // number of participants

int<lower=1> M_2; // number of participant level coefficients

int<lower=1> J_2[N]; // participant indicator

vector[N] Z_2_1;//Participant level predictor values

int<lower=1> N_3; // number of intervention groups

int<lower=1> M_3; // number of intervention group level coefficients

int<lower=1> J_3[N]; // intervention groups indicator

vector[N] Z_3_1; //intervention groups level predictor values

//predictive data

int<lower=1> N_tilde;

matrix[N_tilde, (K-1)] X_tilde;

}

transformed data {

int Kc = K - 1;

matrix[N, Kc] Xc; // centered version of X without an intercept

vector[Kc] means_X; // column means of X before centering

for (i in 2:K) {

means_X[i - 1] = mean(X[, i]);

Xc[, i - 1] = X[, i] - means_X[i - 1];

}

}

parameters {

vector[Kc] b; // fixed effects

real Intercept; // intercept for centered predictors

real<lower=0> sigma; // residual SD

vector<lower=0>[M_1] sd_1; // site level standard deviations

vector[N_1] z_1[M_1]; // standardized site level effects

vector<lower=0>[M_2] sd_2; // participant level standard deviations

vector[N_2] z_2[M_2]; // standardized participant level effects

vector<lower=0>[M_3] sd_3; // intervention groups level standard deviations

vector[N_3] z_3[M_3]; // standardized intervention groups level effects

}

transformed parameters {

vector[N_1] r_1_1; // actual site-level effects

vector[N_2] r_2_1; // actual participant-level effects

vector[N_3] r_3_1; // actual intervention group-level effects

vector[N] mu = Intercept + rep_vector(0.0, N);

r_1_1 = (sd_1[1] * (z_1[1]));

r_2_1 = (sd_2[1] * (z_2[1]));

r_3_1 = (sd_3[1] * (z_3[1]));

for (n in 1:N) {

if (X[n, 2] == 0 && X[n,3]==0) mu[n] += r_1_1[J_1[n]] * Z_1_1[n] + r_3_1[J_3[n]] * Z_3_1[n];

else mu[n] += r_1_1[J_1[n]] * Z_1_1[n] + r_2_1[J_2[n]] * Z_2_1[n] + r_3_1[J_3[n]] * Z_3_1[n];

}

}

model {

// priors including all constants

target += normal_id_glm_lpdf(Y | Xc, mu, b, sigma);

target += normal_lpdf(sigma | 0,10);

target += normal_lpdf(sd_1 | 0,10);

target += std_normal_lpdf(z_1[1]);

target += normal_lpdf(sd_2 | 0,10);

target += std_normal_lpdf(z_2[1]);

target += normal_lpdf(sd_3 | 0,10);

target += std_normal_lpdf(z_3[1]);

}

generated quantities {

// actual population-level intercept

real b_Intercept = Intercept - dot_product(means_X, b);

vector[N_tilde] y_tilde ;

vector [N_tilde] prob_5;

vector [N_tilde] prob_10;

vector [N] log_lik;

for (i in 1:N) {

log_lik[i] =normal_lpdf(Y[i] |mu[i]+Xc[i]*b, sigma);

}

for (i in 1:N_tilde) {

y_tilde[i]=normal_rng(b_Intercept+X_tilde[i]*b, sqrt(sd_1[1]^2+sd_2[1]^2+sigma^2));

prob_5[i] = normal_cdf(-5, b_Intercept+X_tilde[i]*b, sqrt(sd_1[1]^2+sd_2[1]^2+sigma^2));

prob_10[i] = normal_cdf(-10, b_Intercept+X_tilde[i]*b, sqrt(sd_1[1]^2+sd_2[1]^2+sigma^2));

}

}
